# A Short Account of the Annual Report Made to the National Institute of France, on the Subjects of Physiology, Medicine, and Surgery

**Published:** 1810-06

**Authors:** 


### *A short Account of the Annual Report made to the National Institute of France, on the Subjects of Physiology, Medicine, and Surgery.*

### PHYSIOLOGY.

PHYSIOLOGICAL experiments, of all others, are those which require most leisure and patience, while the rigorous exactitude so important and necessary in the sciences is more difficult of attainment in physiology than in any other branch of experimental philosophy. Humboldt, however, while occupied on a voyage, in which obstacles and dangers were daily increasing, directed his attention to some delicate experiments on several of the phenomena of life. He has communicated the researches which he made in America on the respiration of the crocodile with the sharp beak; he was led to ascertain, "that this animal, notwithstanding the volume of its branchiæ and the structure of its pulmonary celluli, suffers in an air which is not renewed; that its respiration is very slow; in the space of an hour and forty-three minutes, a young crocodile, three decimetres in length, took up only about twenty cubic centiemes of oxygen from the surrounding atmosphere."

Since his return to France, M. Humboldt, in conjunction with M. Provencal, has made some additional inquiries into the respiration of fishes. The experiments of these gentlemen, which are numerous, and remarkable for their accuracy, have led them to very important results.

The experiments of Spallanzani and of our colleague, had demonstrated, that it is not by decomposing water that fishes breathe, as some naturalists thought, but by taking up the oxygen dissolved in this liquid, or by coming to the surface of the water, they collect it immediately from the atmosphere. To these observations all our knowledge on the subject was confined; the nature and quantity of the gases had not been established, which were absorbed by these animals in the act of respiration, and the results of these phenomena. The chief object of the experiments of Humboldt and Provencal is to elucidate these questions. With this view they first consider fishes in their natural state and respiring river waters; they next examine the action of the branchiæ in the ambient water impregnated with oxygen and azote, carbonic acid, or a mixture of hydrogen and oxygen, and they afterwards treat of the changes changes produced by fishes in different aeriform fluids in which they are plunged.

Seven tench (ciprinus tinca) were placed under a bell-glass filled with river water, and which contained 4000 cubic centimetrés: after eight hours and a half's respiration the fishes were withdrawn, and the analysis of the air still remaining in the water, shewed that in this space of time the fishes had absorbed 145,4 of oxygen, 57,6 of azote; and that 132 of carbonic acid had been produced; hence it results, as observed by our authors, "that by the respiration of the fishes submitted to this experiment, the volume of the oxygen absorbed exceeded only by two-thirds the volume of the azote which had disappeared, and that no more than one-eighth of the former had been converted into carbonic acid."

Fishes suffer greatly in water entirely freed of air, and after twenty minutes they fall motionless to the bottom of the vessels. In pure oxygen, these animals seem to respire with avidity, and open their branchiæ more widely. In azote and hydrogen, they keep their branchiæ close; seem to dread the contact of these gases, and die very soon after having been plunged into the water which contains them. Carbonic acid kills them in a few minutes; but fishes do not absorb oxygen and azote by their branchiæ alone: the whole surface of their bodies has the faculty of acting on these gases, and of assimilating them. After having withdrawn the fishes from the water saturated with the deleterious gases, and analysed it, some portion of carbonic acid was found in the liquid; but as there had been no oxygen absorbed, it is probable, as observed by Messrs. Humboldt and Provencal, that it was not the result of respiration, but that it had been exhaled from the surface of the body. Such are the principal points in this work, which besides contains other useful observations, and interesting views on the physiology of fishes, but which the limits of this report do not permit us to enter upon.

In speaking of respiration, however, we cannot pass over in silence a memoir read to the Class by M. Provencal, on the respiration of mammiferæ, in which the nerves of the eighth pair had been cut. We have already spoken of the experiments which had been made to ascertain the influence of these nerves on respiration, by which this influence was demonstrated: some doubts however remained as to the way in which it is exercised. M. Provencal was anxious to ascertain if the animal in which the eighth pair of of nerves had been cut, absorbed as much oxygen, and produced the same quantity of carbonic acid before as after the operation. Numerous experiments made with care, proved that the animal after the section of the nerves, absorbed less oxygen, and produced less carbonic acid than before this section; but these changes are produced by gradation only. At first the respiration does not appear weakened; subsequently, it is effected with less strength. Finally, these phenomena cease entirely, but most probably in consequence of the cessation of the mechanical functions of the chest. It was interesting to ascertain if the animal heat diminished in the same proportion with respiration. M. Provencal, therefore, made all the experiments necessary for resolving this question; and it would seem that in fact, the temperature diminishes soon after the nerves have been cut, and the respiration has slackened.

The functions of the organs whose action has been just mentioned are well known; but there exists in animals a certain number of other organs, whose functions are not evident, and as to the use of which physiologists are still divided in opinion. Among this number is the swimming bladder of fishes. This singular organ, which is only to be found in this class of animals, is not met with in all the species, and it exhibits so many varieties in its organization, that at the first glance, one would incline to think, that it did not perform the same functions with some that it did with others. Generally, this bladder is filled with air, and composed of two membranes. Sometimes it communicates with the stomach by a canal: at other times it has no apparent communication; and in this case it contains a peculiar organ of a red colour, and of a lamellated structure, according to the observations of M. Duverna. There are some bladders, however, which are furnished with these red bodies, and which have a canal of communication, and some, but few in number, have peculiar muscles. The opinions of authors vary as to the use of this organ, and of its different parts: in general, it has been thought that it was employed to change the specific gravity of fishes; and that for this purpose, the animal, by means of its muscles, compressed this organ, and thus varied its dimensions, according as it was desirous to remain in equilibrium, to ascend or descend in the medium in which it exists. As to the way in which the air gets admission, it has been thought that it was by means of the canal in such bladders as are furnished with it, and by by means of the glands by secretion in those which have no communication externally. Besides, we know from the experiments of M. Biot, that this air is a mixture of oxygen and azote, and that its nature varies according to the depth at which the fish lives; so that the species which are procured from the bottom of the sea, contain a greater proportion of oxygen, while those which are found near the surface yield more azote. M. Delaroche having collected a great number of fishes in the Mediterranean, has described their swimming bladders, and made us acquainted with some which were unknown before; he has confirmed the experiments of M. Biot; and as to the uses of this organ, he adopts nearly the same results with preceding naturalists.

This air bladder has also been the subject of some researches by Messrs. Humboldt and Provencal. They wished to ascertain what are the relations between this organ and respiration. The chief results of their experiments are as follow: the air contained in the swimming bladder does not depend on the air brought into contact with the branchiæ; the absence of this organ does not affect the process of respiration, but it seems to prevent the production of the carbonic acid gas. Lastly, they have seen tench, from which the swimming bladder had been taken, swim about, rise and sink in water, with as much facility as those which are furnished with them.

These labours have given rise to a very detailed report of M. Cuvier, in which he brings to view all the inquiries which have been undertaken as to the swimming bladder of fishes, and in which he again takes notice of the various questions which have arisen on the subject. After a profound discussion, he arrives at some general results which we have mentioned above, and details every thing which still remains doubtful on the subject.

There are some other experiments from which physiologists may derive great advantage. These relate to the action exercised by substances of various kinds on the bodies of animals, when introduced into the circulation. Medicine, indeed, affords many experiments of this description; but they are still too few in comparison with those which might have been tried.

Messrs. Majendie and Delisle have communicated to the Class their experiments made on animals by means of the matter with which the natives of the isles of Java and of Borneo poison their arrows. This substance is extracted from the *upas tiente*, a plant resembling the *apocineæ*. The experiments experiments of these young physicians have been numerous, and most of them were made on dogs. Whether this poison was introduced into the body of the animal by the blood vessels or by the absorbents; whether it has been poured into wounds, or into the intestines, the same phenomena took place: the animals died in general convulsions. This substance seems to act particularly on the spinal marrow, and to penetrate into the body by means of the circulation; it seems to act but feebly on the brain, and it thus furnishes a proof, that there exists between these two essential parts of the nervous system an independence which anatomy has not demonstrated.

M. Vauquelin has also made some experiments of this kind at the end of his chemical analysis of the juice of the belladonna; he speaks of the effects of this substance on animals. Those which he forced to swallow it, fell down as if intoxicated, in a delirium, precisely similar to that produced by opium.

M. Sage has reported, on the same subject, some more experiments which chance threw in his way, or which he collected from authors, and which confirm the action of this juice on the nervous system, and particularly on the brain.

A young practitioner in medicine, whose name has been mentioned in former annual reports, M. Nysten, has attempted to ascertain the effects of different gases injected into the blood vessels of animals; he used the greater part of the gases with which we are acquainted; atmospheric air, oxygen gas, the oxidulated azotic, carbonic acid, carbonic, phosphorated and hydrogenated gases, &c. are in no respect deleterious. The oxy-muriatic, nitrous acid, and ammoniacal gases, seem to act by very violently irritating the right auricle and the pulmonary ventricle. The sulphurated hydrogen, oxyde of azote, and azotic gases, injure the contractile power of these parts; others also change the nature of the blood so completely, that respiration can no longer convert it from veinous blood into arterial, &c.

### MEDICINE AND SURGERY.

M. Desessartz has communicated the history of an epidemic disease, which raged in three adjoining villages at the same time. Although generally depending on the inclemency of the weather, and on the bad quality of the fruits of the season, this epidemic presented a sensible variety in the nature and intensity of the symptoms, which necessarily necessarily gave rise to essential modifications in the treatment. He shews that the differences depended on the exposure peculiar to each of these villages, on the quality of their respective soils, productions, and way of life of the inhabitants.

M. Sage has presented to the Class, some reflections on the best means of remedying the bite of the viper, and a description of the effects of the poison of the tarantula, with the means employed in Spain for remedying it. All these remedies consist in the internal and external use of the volatile alkali.

M. Tenon continues to enrich the art of surgery. He has communicated to the Class three Memoirs, one on the exfoliation of the bones, another on the operation of trepaning the cranium, and a third on some kinds of hernia. In the first, he inquires if the bones of the great extremities of the body are exfoliated after amputation; and it results from his numerous experiments on dogs, hares, and sheep, that after all amputations, the denuded extremity of the long bones is exfoliated, as also happens to the flat bones when laid bare, before being covered with a cicatrix. In the second, he gives the description of all the phænomena which took place in the cure of a wound in the head, in consequence of which, the trepan was resorted to; and which was cured after 151 days treatment.

In the third, he describes an ingenious method resorted to by himself; for the reduction of two crural hernia, and gives some observations on the operation for an inguinal hernia. In order to succeed in the reduction of these crural hernia, "I directed an assistant to get upon the bed of the patient, and place himself between the knees of the latter, making him raise them as high as possible; the pillows being withdrawn, I employed another person to hold the leg and foot on the ruptured side, and to turn strongly inwards the great toe as well as the knee and thigh." When matters were thus arranged, M. Tenon succeeded by degrees in returning the intestines into the abdomen, so that the patient had no occasion for any operation.

M. Pelletan has communicated to us some interesting observations on aneurisms, and on the chirurgical operations which these diseases require.

M. Larrey has submitted to the Class, a Memoir, on which a Report has been made, and which suggests that in cases of gun-shot wounds, followed by gangrene, that we should not expect to put a stop to the gangrene by performing amputation.

